# Supplementary material for: Extrapulmonary tuberculosis in Pakistan- A nation-wide multicenter retrospective study
Source: PLoS One. 2020 Apr 28;15(4):e0232134. doi: 10.1371/journal.pone.0232134 (PMC7188211; doi:10.1371/journal.pone.0232134)
Supplement: S1 Fig — (PDF) [file pone.0232134.s001.pdf]

Support document

S1 Figure: Pakistan map showing provincial and regional boundaries

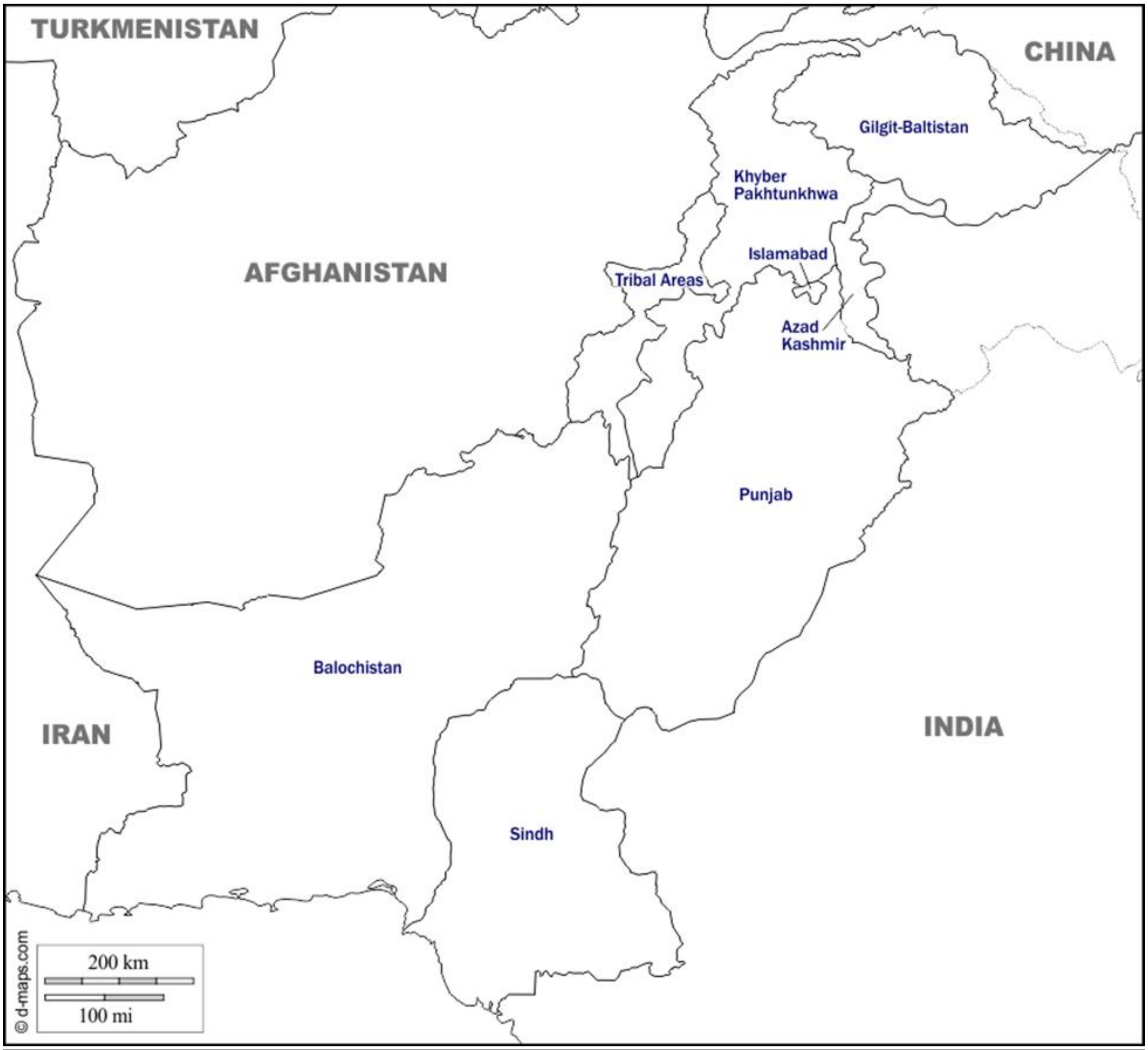

Source ; Pakistan – provinces Azad Kashmir, Balochistan, Gilgit- Baltistan, Islamabad, Khyber Pakhtunkhwa, Punjab, Sindh, Tribal Areas.
https://d-maps.com/m/asia/pakistan/pakistan/pakistan18.gif
