# Supplementary material for: Extrapulmonary tuberculosis in Pakistan- A nation-wide multicenter retrospective study
Source: PLoS One. 2020 Apr 28;15(4):e0232134. doi: 10.1371/journal.pone.0232134 (PMC7188211; doi:10.1371/journal.pone.0232134)
Supplement: S1 Table — EPTB–Extra pulmonary tuberculosis, PTB-Pulmonary tuberculosis, HCF-Health care facility, HCP-Health care provider, Pub-public, Pvt–private, THC-Tertiary health care, SHC–Secondary health Care, PHC-Primary health care, GP–General Practitioner, GX- GeneXpert, HP- histopathology, PR- Paper register, ER- electronic file, PJB-Punjab, SND- Sind, KP-Khyber Pakhtunkhwa, BTN-Balochistan, AJK -Azad Jammu Kashmir, GB-Gilgit Baltistan, FATA- Federally administered tribal area, ICT-Islamabad capital Territory. (PDF) [file pone.0232134.s002.pdf]

**S1 Table: Characteristics of the selected health care facilities and TB cases notified in 2016**

| | Province | Health Care facility | | | | Diagnostic Facility | | TB-Register | ALL TB Cases (n) | Total PTB | Extra Pulmonary TB | | | |
|---|---|---|---|---|---|---|---|---|---|---|---|---|---|---|
| | | Code | District | Health care provider | Health care Tier | Gxpert | H/P | | | | (n) | % | Site specified | |
| | | | | | | | | | | | | | (n) | (%) |
| 1 | PJB | p-1 | Lahore | Public | THC | Yes | Yes | Paper | 1523 | 510 | 1013 | 66.5% | 768 | 75.8% |
| 2 | PJB | p-2 | Sargodha | Public | SHC | Yes | No | Paper | 2022 | 1444 | 578 | 28.6% | 536 | 92.7% |
| 3 | PJB | p-3 | Lahore | Public | PHC | No | No | Paper | 840 | 636 | 204 | 24.3% | 172 | 84.3% |
| 4 | PJB | p-4 | Sahiwal | Public | PHC | No | No | Paper | 251 | 173 | 78 | 31.1% | 74 | 94.9% |
| 5 | PJB | p-5 | Lahore | Private | THC | Yes | Yes | Elect | 7253 | 6102 | 1151 | 15.9% | 1149 | 99.8% |
| 6 | PJB | p-6 | Bahawalnagar | Private | GP | No | No | Elect | 1122 | 956 | 166 | 14.8% | 166 | 100.0% |
| 7 | PJB | p-7 | Bahawalpur | Private | GP | No | No | Elect | 426 | 408 | 18 | 4.2% | 18 | 100.0% |
| 8 | PJB | p-8 | Faisalabad | Private | GP | No | No | Elect | 1981 | 1482 | 499 | 25.2% | 498 | 99.8% |
| 9 | PJB | p-9 | Gujrat | Private | GP | No | No | Elect | 226 | 215 | 11 | 4.9% | 11 | 100.0% |
| 10 | PJB | p-10 | Lahore | Private | GP | No | No | Elect | 3288 | 2409 | 879 | 26.7% | 807 | 91.8% |
| 11 | PJB | p-11 | Muzaffargarh | Private | GP | No | No | Elect | 1197 | 767 | 430 | 35.9% | 430 | 100.0% |
| 12 | PJB | p-12 | Rawalpindi | Private | GP | No | No | Elect | 979 | 694 | 285 | 29.1% | 280 | 98.2% |
| 13 | PJB | p-13 | R.Y. Khan | Private | GP | No | No | Elect | 1634 | 1472 | 162 | 9.9% | 158 | 97.5% |
| 14 | PJB | p-14 | Shaikhupura | Private | GP | No | No | Elect | 788 | 682 | 106 | 13.5% | 50 | 47.2% |
| 15 | SND | S-1 | Larkana | Public | THC | Yes | Yes | Paper | 1169 | 802 | 367 | 31.4% | 298 | 81.2% |
| 16 | SND | S-2 | Karachi | Public | THC | Yes | Yes | Paper | 1145 | 839 | 306 | 26.7% | 301 | 98.4% |
| 17 | SND | S-3 | Jamshoro | Public | SHC | Yes | NO | Paper | 4036 | 3425 | 611 | 15.1% | 610 | 99.8% |
| 18 | SND | S-4 | Hyderabad | Public | SHC | No | NO | Paper | 358 | 172 | 186 | 52.0% | 186 | 100.0% |
| 19 | SND | S-5 | Khairpur | Public | SHC | No | NO | Paper | 1450 | 1201 | 249 | 17.2% | 236 | 94.8% |
| 20 | SND | S-6 | Karachi | Public | PHC | Yes | NO | Paper | 882 | 735 | 147 | 16.7% | 144 | 98.0% |
| 21 | SND | S-7 | Khairpur | Public | PHC | No | NO | Paper | 239 | 188 | 51 | 21.3% | 51 | 100.0% |
| 22 | SND | S-8 | Karachi | Private | THC | Yes | Yes | Paper | 2688 | 2041 | 647 | 24.1% | 644 | 99.5% |
| 23 | SND | S-9 | Karachi | Private | GP | No | NO | Excel | 2722 | 1947 | 775 | 28.5% | 769 | 99.2% |
| 24 | SND | S-10 | Hyderabad | Private | GP | No | NO | Excel | 439 | 359 | 80 | 18.2% | 80 | 100.0% |
| 25 | SND | S-11 | Sukker | Private | GP | No | NO | Excel | 680 | 585 | 95 | 14.0% | 94 | 98.9% |
| 26 | KP | K-1 | Peshawar | Public | THC | Yes | Yes | Paper | 256 | 110 | 146 | 57.0% | 138 | 94.5% |
| 27 | KP | K-2 | Mansehra | Public | THC | No | NO | Excel | 423 | 262 | 161 | 38.1% | 160 | 99.4% |
| 28 | KP | K-3 | Peshawar | Public | SHC | Yes | NO | Excel | 1830 | 808 | 1022 | 55.8% | 729 | 71.3% |
| 29 | KP | K-4 | Mardan | Public | SHC | Yes | NO | Paper | 2035 | 881 | 1154 | 56.7% | 1037 | 89.9% |
| 30 | KP | K-5 | Mardan | Public | PHC | No | NO | Excel | 268 | 173 | 95 | 35.4% | 80 | 84.2% |
| 31 | KP | K-6 | Peshawar | Public | PHC | No | NO | Paper | 211 | 139 | 72 | 34.1% | 71 | 98.6% |

| | | | | | | | | | | | | | | |
|---|---|---|---|---|---|---|---|---|---|---|---|---|---|---|
| 32 | KP | K-7 | Peshawar | Private | GP | No | NO | Excel | 2779 | 1499 | 1280 | 46.1% | 1261 | 98.5% |
| 33 | BTN | B-1 | Quetta | Public | THC | No | Yes | Paper | 626 | 227 | 399 | 63.7% | 394 | 98.7% |
| 34 | BTN | B-2 | K.Abdullah | Public | SHC | No | NO | Paper | 600 | 480 | 120 | 20.0% | 112 | 93.3% |
| 35 | BTN | B-3 | Jafferabad | Public | SHC | No | NO | Paper | 343 | 246 | 97 | 28.3% | 1 | 1.0% |
| 36 | BTN | B-4 | Zhob | Public | PHC | No | NO | Paper | 542 | 357 | 185 | 34.1% | 90 | 48.6% |
| 47 | FATA | F-1 | Bajour | Public | PHC | Yes | No | Paper | 650 | 336 | 314 | 48.3% | 307 | 97.8% |
| 48 | FATA | F-2 | Khyber agency | Public | PHC | Yes | No | Paper | 288 | 130 | 158 | 54.9% | 158 | 100.0% |
| 42 | GB | G-1 | Skardu | Public | SHC | No | No | Paper | 120 | 83 | 37 | 30.8% | 34 | 91.9% |
| 43 | GB | G-2 | Skardu | Public | PHC | No | No | Paper | 339 | 226 | 113 | 33.3% | 111 | 98.2% |
| 44 | GB | G-3 | Gligit | Public | PHC | No | No | Paper | 347 | 227 | 120 | 34.6% | 120 | 100.0% |
| 45 | GB | G-4 | Tangir | Public | PHC | No | No | Paper | 234 | 178 | 56 | 23.9% | 56 | 100.0% |
| 46 | GB | G-5 | Challas | Public | PHC | No | No | Paper | 653 | 542 | 111 | 17.0% | 111 | 100.0% |
| 37 | AJK | A-1 | Muzaffrabad | Public | THC | Yes | Yes | Paper | 188 | 104 | 84 | 44.7% | 78 | 92.9% |
| 38 | AJK | A-2 | Kotli | Public | SHC | No | NO | Paper | 174 | 114 | 60 | 34.5% | 60 | 100.0% |
| 39 | AJK | A-3 | Mirpur | Public | SHC | No | NO | Paper | 179 | 124 | 55 | 30.7% | 54 | 98.2% |
| 41 | AJK | A-4 | Muzaffrabad | Public | PHC | No | NO | Paper | 310 | 177 | 133 | 42.9% | 131 | 98.5% |
| 40 | AJK | A-5 | Mirpur | Public | PHC | No | No | Paper | 73 | 58 | 15 | 20.5% | 15 | 100.0% |
| 49 | ICT | I-1 | ICT | Public | THC | No | Yes | Paper | 1256 | 555 | 701 | 55.8% | 605 | 86.3% |
| 50 | ICT | I-2 | ICT | Public | PHC | No | No | Paper | 30 | 22 | 8 | 26.7% | 8 | 100.0% |
| | | | | | | | | | | | | | | |
| **Provincial/Regional summary** | | | | | | | | | | | | | | |
| Province /Regions | | Study sites | Districts (n) | HCP | HC-Tier | Diagnostic Facility | | TB register | All TB | Total PTB | Extrapulmonary TB | | | |
| | | | | Pub+Pvt | P+S+T | GX | HP | P+E | | | (n) | (%) | Site specified | |
| PJB | | 14 | 10 | 4+10 | 11+1+2 | 3 | 2 | 4+10 | 23530 | 17950 | 5580 | 23.7% | 5117 | 91.7% |
| SND | | 11 | 6 | 7+4 | 05+3+3 | 5 | 3 | 8+03 | 15808 | 12294 | 3514 | 22.2% | 3413 | 97.1% |
| KP | | 7 | 3 | 6+1 | 03+2+2 | 1 | 1 | 3+04 | 7802 | 3872 | 3930 | 50.4% | 3476 | 88.4% |
| BTN | | 4 | 4 | 4+0 | 01+2+1 | 0 | 1 | 4+0 | 2111 | 1310 | 801 | 37.9% | 597 | 74.5% |
| FATA | | 2 | 2 | 2+0 | 02+0+0 | 2 | 0 | 2+0 | 938 | 466 | 472 | 50.3% | 465 | 98.5% |
| GB | | 5 | 3 | 5+0 | 04+1+0 | 0 | 0 | 5+0 | 1693 | 1256 | 437 | 25.8% | 432 | 98.9% |
| AJK | | 5 | 3 | 5+0 | 02+2+1 | 1 | 1 | 5+0 | 924 | 577 | 347 | 37.6% | 338 | 97.4% |
| ICT | | 2 | 1 | 2+0 | 01+0+1 | 0 | 1 | 2+0 | 1286 | 577 | 709 | 55.1% | 613 | 86.5% |
| **G.total** | | **50** | **32** | **35+15** | **29+11+10** | **12** | **1** | **33+17** | **54092** | **38302** | **15790** | **29.2%** | **14451** | **91.5%** |

EPTB – Extra pulmonary tuberculosis, PTB-Pulmonary tuberculosis, HCF-Health care facility , HCP-Health care provider, Pub-public , Pvt – private , THC-Tertiary health care, SHC –Secondary health Care, PHC-Primary health care, GP –General Practitioner, GX- GeneXpert , HP- histopathology , PR- Paper register , ER- electronic file , PJB-Punjab, SND- Sind, KP-Khyber Pakhtunkhwa, BTN-Balochistan, AJK -Azad Jammu Kashmir, GB-Gilgit Baltistan, FATA- Federally administered tribal area, ICT-Islamabad capital Territory
