# Supplementary material for: Extrapulmonary tuberculosis in Pakistan- A nation-wide multicenter retrospective study
Source: PLoS One. 2020 Apr 28;15(4):e0232134. doi: 10.1371/journal.pone.0232134 (PMC7188211; doi:10.1371/journal.pone.0232134)
Supplement: S2 Table — PTB- Pulmonary tuberculosis, EPTB–Extra pulmonary tuberculosis, N = New TB case, R = Relapse TB case, GB–Gilgit Baltistan, KPK–Khyber Pakhtunkhwa, AJK-Azad Jamu Kashmir, FATA- Federally administered tribal area, ICT–Islamabad capital Territory, GP- General practioner. (PDF) [file pone.0232134.s003.pdf]

## S2 Table: National and study sites TB case notifications by province and region in 2016

| | Pakistan | PJB | SND | KP | BTN | FATA | GB | AJK | ICT |
|---|---|---|---|---|---|---|---|---|---|
| Population (Million) 2016 | 189.1 | 97.9 | 43.4 | 25.2 | 9.9 | 4.4 | 1.2 | 5.2 | 1.15 |
| **TB cases notified Nationally in 2016** | | | | | | | | | |
| All TB cases | 366061 | 221739 | 72830 | 45457 | 10462 | 4379 | 2995 | 5831 | 2368 |
| All PTB cases | 293100 | 185985 | 60026 | 28623 | 7903 | 2909 | 2247 | 4227 | 1180 |
| All EPTB cases | 72961 | 35754 | 12804 | 16834 | 2559 | 1470 | 748 | 1604 | 1188 |
| % EPTB | **19.9% (19.8-20.1)** | **16.1% (16.0-16.3)** | **17.6% (17.3-17.9)** | **37.0% (36.6-37.5)** | **24.5% (23.6-25.3)** | **33.6% (32.2-35.0)** | **25.0% (23.4-26.6)** | **27.5% (26.4-28.7)** | **50.2% (48.1-52.2)** |
| **TB cases notified by study sites in 2016** | | | | | | | | | |
| All TB cases | 54092 | 23530 | 15808 | 7802 | 2111 | 938 | 1693 | 924 | 1286 |
| All PTB cases | 38302 | 17950 | 12294 | 3872 | 1310 | 466 | 1256 | 577 | 577 |
| All EPTB cases | 15790 | 5580 | 3514 | 3930 | 801 | 472 | 437 | 347 | 709 |
| % EPTB | **29.2%** | **23.7%** | **22.2%** | **50.4%** | **37.9%** | **50.3%** | **25.8%** | **37.6%** | **55.1%** |
| **Proportion of notified TB cases included in study sample** | | | | | | | | | |
| % TB cases | 14.8% | 10.6% | 21.7% | 17.2% | 20.2% | 21.4% | 56.5% | 15.8% | 54.3% |
| % PTB cases | 13.1% | 9.7% | 20.5% | 13.5% | 16.6% | 16.0% | 55.9% | 13.7% | 48.9% |
| % EPTB cases | 21.6% | 15.6% | 27.4% | 23.3% | 31.3% | 32.1% | 58.4% | 21.6% | 59.7% |
| **Health care facilities sampled *** | | | | | | | | | |
| PHC (Level-I) | 16/810 | 2/371 | 2/134 | 2/126 | 1/119 | 2/10 | 4/13 | 2/37 | 1/4 |
| PHC (Dist.GP clusters)* | 13/92 | 13/36 | 3/20 | 1/18 | 0/9 | 0/0 | 0/3 | 0/5 | 0/1 |
| SHC (Level-II) | 11/397 | 1/151 | 3/84 | 2/90 | 2/32 | 0 | 1/13 | 2/9 | 0/4 |
| THC(level-III) | 10/46 | 2/20 | 3/12 | 2/9 | 1/3 | 0 | 0 | 1/1 | 1/1 |
| Total | 50/1345 | 18/378 | 11/250 | 7/243 | 4/163 | 2/10 | 5/29 | 5/52 | 2/10 |
| *Data is number of health facilities sampled / total number of reporting health facilities in 2016 | | | | | | | | | |

PTB- Pulmonary tuberculosis , EPTB – Extra pulmonary tuberculosis, N=New TB case , R=Relapse TB case, GB – Gilgit Baltistan, KPK – Khyber Pakhtunkhwa, AJK-Azad Jamu Kashmir, FATA- Federally administered tribal area, ICT – Islamabad capital Territory , GP- General practioner .
