# Supplementary material for: Extrapulmonary tuberculosis in Pakistan- A nation-wide multicenter retrospective study
Source: PLoS One. 2020 Apr 28;15(4):e0232134. doi: 10.1371/journal.pone.0232134 (PMC7188211; doi:10.1371/journal.pone.0232134)
Supplement: S3 Table — F-EPTB = Female Extrapulmonary TB, F-PTB = Female Pulmonary TB cases, M-EPTB = Male Extrapulmonary TB, M-PTB = Male Pulmonary TB cases. (PDF) [file pone.0232134.s004.pdf]

S3 Table: Age and sex specific pulmonary and extrapulmonary tuberculosis notifications and odds of female (OR) for having extrapulmonary tuberculosis by place of residence.

	F EPTB	F PTB	M EPTB	M PTB	OR	95% CI	F EPTB	F PTB	M EPTB	M PTB	OR	95% CI	F EPTB	F PTB	M EPTB	M PTB	OR	95% CI
	**All STUDY SITES**						**PUNJAB**						**SINDH**					
**0-14**	1835	2501	1772	2230	0.92	0.85-1.00	318	525	184	257	0.85	0.67-1.07	249	1074	155	968	1.45	1.16-1.80
**15-24**	2597	5714	1877	4218	1.02	0.95-1.1	1073	2683	817	1999	0.98	0.88-1.09	775	1819	425	1326	1.33	1.16-1.53
**25-34**	1628	3240	1246	3234	1.30	1.19-1.42	657	1538	551	1577	1.22	1.07-1.39	465	1031	303	1158	1.72	1.46-2.04
**35-44**	808	2195	841	2525	1.11	0.98-1.23	319	1176	379	1379	0.99	0.83-1.17	198	626	212	839	1.25	1.00-1.56
**45-54**	625	2152	726	2794	1.12	0.99-1.26	253	1199	311	1525	1.03	0.86-1.24	146	555	194	940	1.27	1.00-1.62
**55-64**	440	1686	518	2389	1.20	1.04-1.38	183	916	191	1218	1.27	1.02-1.59	89	389	147	780	1.21	0.91-1.62
**65+**	335	1355	539	2050	0.94	0.80-1.09	135	778	207	1164	0.98	0.77-1.23	61	269	95	520	1.24	0.87-1.77
	**KHYBER PAKHTUNKHWA**						**BALOCHISTAN**						**AZAD JAMMU KASHMIR**					
**0-14**	868	381	981	353	0.82	0.69-0.97	185	234	191	260	1.08	0.82-1.41	19	29	15	20	0.87	0.36-2.12
**15-24**	420	698	378	571	0.91	0.76-1.09	79	114	58	58	0.69	0.44-1.10	41	93	42	49	0.51	0.29-0.89
**25-34**	267	296	198	276	1.26	0.98-1.61	54	132	40	50	0.51	0.30-0.86	35	42	34	38	0.93	0.49-1.77
**35-44**	143	175	119	153	1.05	0.76-1.46	35	56	18	36	1.25	0.62-2.53	23	30	25	31	0.95	0.45-2.03
**45-54**	131	200	95	146	1.01	0.72-1.41	20	57	21	44	0.74	0.36-1.52	21	29	21	47	1.62	0.76-3.47
**55-64**	91	173	81	181	1.18	0.82-1.69	20	87	20	63	0.72	0.36-1.46	10	30	16	42	0.88	0.35-2.19
**65+**	61	121	96	148	0.78	0.52-1.16	22	54	38	65	0.70	0.37-1.32	15	49	30	48	0.49	0.23-1.02
	**GILGIT BALTISTAN**						**FATA**						**ISLAMABAD CAPITAL TERRITORY**					
**0-14**	89	213	103	330	1.34	0.96-1.87	83	30	132	37	0.79	0.45-1.35	24	15	11	5	1.34	0.96-1.87
**15-24**	63	169	26	80	1.15	0.68-1.95	38	55	34	65	1.32	0.74-2.37	108	83	97	70	0.94	0.62-1.43
**25-34**	40	120	17	23	0.45	0.22-0.93	36	45	26	45	1.38	0.72-2.66	74	36	77	67	1.79	1.07-2.99
**35-44**	19	76	10	20	0.50	0.20-1.24	18	22	22	18	0.67	0.28-1.62	53	34	56	49	1.36	0.77-2.43
**45-54**	11	60	12	19	0.29	0.11-0.76	10	23	19	17	0.39	0.14-1.05	33	29	53	56	1.20	0.64-2.25
**55-64**	12	40	13	40	0.92	0.38-2.27	10	28	11	25	0.81	0.29-2.23	25	23	39	40	1.11	0.54-2.29
**65+**	11	29	11	37	1.28	0.49-3.35	11	31	22	22	0.35	0.14-0.88	19	24	40	46	0.91	0.44-1.90

F-EPTB-Female Extrapulmonary TB , F-PTB- Female Pulmonary TB cases, M-EPTB-Male Extrapulmonary TB , M-PTB-Male Pulmonary TB cases, OR –Odds raio
