# Supplementary material for: Extrapulmonary tuberculosis in Pakistan- A nation-wide multicenter retrospective study
Source: PLoS One. 2020 Apr 28;15(4):e0232134. doi: 10.1371/journal.pone.0232134 (PMC7188211; doi:10.1371/journal.pone.0232134)
Supplement: S4 Table — A: Notified tuberculosis cases and extra-pulmonary manifestations of tuberculosis by sex, age groups and health facilities in Punjab, Pakistan during 2016 EPTB–Extra pulmonary tuberculosis, Site-NOS: EPTB site not specified, LN-EXT-lymphatic extra-thoracic, LN-INT-Lymphatic intra thoracic, ABD-Abdomen, OAS–Osteoarticular spine, OAOS-Osteoarticular other than spine,CNS-Central nervous system, DIS/MIL–Disseminated /Miliary TB. B: Notified tuberculosis cases and extrapulmonary manifestation of tuberculosis by sex, age groups and health facilities in Sindh, Pakistan during 2016 EPTB–Extra pulmonary tuberculosis, Site-NOS: EPTB site not specified, LN-EXT-lymphatic extra-thoracic, LN-INT-Lymphatic intra thoracic, ABD-Abdomen, OAS–Osteoarticular spine, OAOS-Osteoarticular other than spine,CNS-Central nervous system, DIS/MIL–Disseminated /Miliary TB. C: Notified tuberculosis cases and extrapulmonary manifestations of tuberculosis by sex, age groups and health facilities in Khyber Pakhtunkhwa, Pakistan during 2016 EPTB–Extra pulmonary tuberculosis, Site-NOS: EPTB site not specified, LN-EXT-lymphatic extra-thoracic, LN-INT-Lymphatic intra thoracic, ABD-Abdomen, OAS–Osteoarticular spine, OAOS-Osteoarticular other than spine,CNS-Central nervous system, DIS/MIL–Disseminated /Miliary TB. D: Notified tuberculosis cases and extrapulmonary manifestations of tuberculosis by sex, age groups and health facilities in Balochistan, Pakistan, during 2016 EPTB–Extra pulmonary tuberculosis, Site-NOS: EPTB site not specified, LN-EXT-lymphatic extra-thoracic, LN-INT-Lymphatic intra thoracic, ABD-Abdomen, OAS–Osteoarticular spine, OAOS-Osteoarticular other than spine,CNS-Central nervous system, DIS/MIL–Disseminated /Miliary TB. E: Notified tuberculosis cases and extrapulmonary manifestations of tuberculosis by sex, age groups and health facilities in Federally Administered Tribal Areas, Pakistan during 2016 EPTB–Extra pulmonary tuberculosis, Site-NOS: EPTB site not specified, LN-EXT-lymphatic e [file pone.0232134.s005.pdf]

S4A Table: Notified tuberculosis cases and extra-pulmonary manifestations of tuberculosis by sex, age groups and health facilities in Punjab, Pakistan during 2016

	Site-NOS	PLEURAL	LN-EXT	LN-INT	ABD	OAS	OAOS	CNS	DIS/MIL	OTHER	Tot.EPTB	All TB	EPTB%
Total	463 (8.3%)	2019 (36.2%	1305 23.4%)	86 (1.5%)	672 (12.0%)	291 (5.2%)	288 (5.2%)	200 (3.6%)	10 (0.2%)	246 (4.4%)	5580 (100%)	23530	23.7%
Median Age(IQR)	28 (19,45)	30 (20,45)	22 (16,32)	21 (16,31)	25 (18,35)	32 (24,50)	33 (21,50)	30 (18,50)	30 (23,50)	27 (20,40)	27 (19,40)	32 (21,50)	
**TB cases notified by sex**													
Female	241	911	801	51	386	162	148	96	4	140	2940	11759	25.0%
Male	222	1108	504	35	286	129	140	104	6	106	2640	11771	22.4%
F:M	1.1	0.8	1.6	1.5	1.3	1.3	1.1	0.9	0.7	1.3	1.1	1.0	
**TB cases notified by age group**													
0-14	25	107	223	19	68	17	14	10		19	502	1284	39.1%
15-24	168	654	511	32	252	56	76	70	3	68	1890	6572	28.8%
25-34	82	429	274	16	161	78	59	38	2	69	1208	4323	27.9%
35-44	70	254	122	9	88	50	41	19	1	44	698	3253	21.5%
45-54	62	239	81	3	44	37	50	23	3	22	564	3288	17.2%
55-64	26	169	52	5	34	29	27	18	1	13	374	2508	14.9%
65+	30	167	41	2	24	24	21	22		11	342	2284	15.0%
NA			1		1		0				2	18	11.1%
Adults(All)	438	1912	1081	67	603	274	274	190	10	227	5076	22228	22.8%
**TB cases notified by health facility**							0						
P-1	245	170	278		127	23	21	95	8	46	1013	1523	66.5%
P-2	42	191	151	1	51	61	49	7	1	24	578	2022	28.6%
P-3	32	57	31		67	3	7	4		3	204	840	24.3%
P-4	4	10	3		13	19	3			26	78	251	31.1%
P-5	2	604	334	5	42	78	7	29		50	1151	7253	15.9%
P-6		39	40		33		6			48	166	1122	14.8%
P-7		4	1	5	2	2	2			2	18	426	4.2%
P-8	1	172	141		81	10	82	12			499	1981	25.2%
P-9		3	5	1	2		0				11	226	4.9%
P-10	72	238	178	61	142	64	57	27		40	879	3288	26.7%
P-11		357	44		14	15	0				430	1197	35.9%
P-12	5	91	46	8	75	9	24	24		3	285	979	29.1%
P-13	4	82	12	5	18	6	28	2	1	4	162	1634	9.9%
P-14	56	1	41		5	1	2				106	788	13.5%

EPTB – Extra pulmonary tuberculosis, Site-NOS: EPTB site not specified, LN-EXT-lymphatic extra-thoracic, LN-INT-Lymphatic intra thoracic, ABD-Abdomen, OAS – Osteoarticular spine , OAOS-Osteoarticular other than spine ,CNS-Central nervous system, DIS/MIL– Disseminated /Miliary TB

S4B Table: Notified tuberculosis cases and extrapulmonary manifestation of tuberculosis by sex, age groups and health facilities in Sindh, Pakistan during 2016

	Site-NOS	PLEURAL	LN-EXT	LN-INT	ABD	OAS	OAOS	CNS	DIS/MIL	OTHER	All EPTB	All TB	EPTB%
**Total**	101 (2.9%)	1190 (33.9%)	1002 (28.5%)	32 (0.9%)	602 (17.1%)	272 (7.7%)	94 (2.7%)	80 (2.3%)	21 (0.6%)	120 (3.4%)	3514 (100%)	15808	22.2%
**Median Age (IQR)**	25 (18,35)	30 (20,60)	23 (16,32)	28 (18.5,45.5)	22 (16,33)	31 (22,50)	29 (19,45)	20 (15,35.5)	22 (18,32)	28 (19,40)	25 (18,40)	27 (18,45)	
**TB cases notified by sex**													
Female	46	560	666	21	377	152	46	48	10	57	1983	7746	25.6%
Male	55	630	336	11	225	120	48	32	11	63	1531	8062	19.0%
F:M	0.8	0.9	2.0	1.9	1.7	1.3	1.0	1.5	0.9	0.9	1.3	1.0	
**TB cases notified by age group**							0						
0-14	9	58	173	5	107	12	11	18	1	10	404	2446	16.5%
15-24	38	370	372	10	228	74	30	32	11	35	1200	4345	27.6%
25-34	25	262	234	5	125	60	12	9	4	32	768	2957	26.0%
35-44	14	152	111	4	54	40	14	4	3	14	410	1875	21.9%
45-54	10	143	61	4	45	44	14	5		14	340	1835	18.5%
55-64	4	122	33	2	24	19	10	9	1	12	236	1405	16.8%
65+	1	83	18	2	19	23	3	3	1	3	156	945	16.5%
Adult (All)	92	1132	829	27	495	260	83	62	20	110	3110	13362	23.3%
**TB cases notified by health facility**													
S-1	69	70	94		50	30	25	11	8	10	367	1169	31.4%
S-2	5	127	95	2	17	18	4	7	2	29	306	1145	26.7%
S-3	1	286	158		72	46	16	17		15	611	4036	15.1%
S-4		51	86		37	8	0	3	1		186	358	52.0%
S-5	13	76	60	1	63	11	0	5	2	18	249	1450	17.2%
S-6	3	72	30		13	19	3	1	1	5	147	882	16.7%
S-7		29			4		0			18	51	239	21.3%
S-8	3	146	211	13	176	52	10	19	6	11	647	2688	53%
S-9	6	273	228	7	146	54	33	15	1	12	775	2722	28.5%
S-10		28	16	9	19	5	3				80	439	18.2%
S-11	1	32	24		5	29	0	2		2	95	680	14.0%
**Total**	**10**	**479**	**479**	**29**	**346**	**140**	46	**36**	**7**	**25**	**1597**	6529	24.5%

EPTB – Extra pulmonary TB, Site-NOS: EPTB site not specified, LN-EXT-lymphatic extra-thoracic, LN-INT-Lymphatic intra thoracic, ABD-Abdomen, OAS – Osteoarticular spine , OAOS- Osteoarticular other than spine ,CNS-Central nervous system, DIS/MIL – Disseminated /Miliary TB

S4C Table: Notified tuberculosis cases and extrapulmonary manifestations of tuberculosis by sex, age groups and health facilities in Khyber Pakhtunkhwa, Pakistan during 2016

	Site-NOS	PLEURAL	LN-EXT	LN-INT	ABD	OAS	OAOS	CNS	DIS/MIL	OTHER	Tot. EPTB	All TB	EPTB%
**Total**	454 (11.6%)	918 (23.4%)	635 (16.2%)	57 (1.5%)	1350 (34.4%)	163 (4.1%)	101 (2.6%)	90 (2.3%)	30 (0.8%)	132 (3.4%)	3930 (100%)	7802	50.4%
**Median Age (IQR)**	12 (5,24)	20 (10,36)	17 (9,30)	20 (13,27)	10 (4,23)	30 (17,50)	25 (15,45)	16 (8,35)	19 (15,40)	18 (8,30)	16 (6,30)	20 (11,35)	
**TB cases notified by sex**													
Female	241	404	362	30	666	98	48	46	11	76	1982	4026	49.2%
Male	213	514	273	27	684	65	53	44	19	56	1948	3776	51.6%
F:M	1.1	0.8	1.3	1.1	1.0	1.5	0.9	1.0	0.6	1.4	1.0	1.1	
**TB cases notified by age group**							0						
0-14	264	309	274	15	830	32	24	42	7	52	1849	2583	71.6%
15-24	78	237	140	25	201	34	25	15	9	34	798	2067	38.6%
25-34	58	109	90	6	123	29	16	10	3	21	465	1037	44.8%
35-44	19	85	47	4	65	14	8	7	6	7	262	590	44.4%
45-54	16	57	42	3	56	24	15	5	1	7	226	572	39.5%
55-64	13	56	19	2	38	20	8	8		8	172	526	32.7%
65+	6	65	23	2	36	10	5	3	4	3	157	426	36.9%
NA					1		0				1	1	100.0%
Adult (All)	190	609	361	42	519	131	77	48	23	80	2080	5218	39.9%
**TB cases notified by health facility**													
K-1	8	44	30	1	20	11	7	13	3	9	146	256	57.0%
K-2	1	35	34		43	7	5		4	32	161	423	38.1%
K-3	293	145	131	5	301	34	13	17	15	68	1022	1830	55.8%
K-4	117	182	186		545	44	37	24	1	18	1154	2035	56.7%
K-5	15	23	12		31	3	9	2			95	268	35.4%
K-6	1	23	17		13	10	2	1		5	72	211	34.1%
K-7	19	466	225	51	397	54	28	33	7		1280	2779	46.1%

EPTB – Extra pulmonary tuberculosis, Site-NOS: EPTB site not specified, LN-EXT-lymphatic extra-thoracic, LN-INT-Lymphatic intra thoracic, ABD-Abdomen, OAS – Osteoarticular spine , OAOS- Osteoarticular other than spine ,CNS-Central nervous system, DIS/MIL – Disseminated /Miliary TB

S4D Table: Notified tuberculosis cases and extrapulmonary manifestations of tuberculosis by sex, age groups and health facilities in Balochistan, Pakistan, during 2016

	Site-NOS	PLEURAL	LN-EXT	LN-INT	ABD	OAS	OAOS	CNS	DIS/MIL	OTHER	Tot. EPTB	All TB	EPTB%
Total	204 (25.5%)	83 (10.4%)	59 (7.4%)	5 (0.6%)	124 (15.5%)	56 (7.0%)	37 (4.6%)	205 (25.6%)	12 (1.5%)	16 (2.0%)	801 100%	2111	37.9%
Median Age (IQR)	7 (2,25)	24 (19,40)	16 (10,23)	8 (8,20)	15.5 (3.5,30)	38.5 (25,60)	30 (12,40)	8 (2,23)	21 (15.5,46.5)	23.5 (8,38.5)	16 (4,32)	20 (4,40)	
**TB cases notified by sex**													
Female	114	30	38	2	69	27	19	98	8	10	415	1149	36.1%
Male	90	53	21	3	55	29	18	107	4	6	386	962	40.1%
F:M	1.3	0.6	1.8	0.7	1.3	0.9	1.1	0.9	2.0	1.7	1.1	1.2	
**TB cases notified by age group**													
0-14	127	11	23	2	59	9	11	126	3	5	376	870	43.2%
15-24	22	31	18	2	19	4	4	29	4	4	137	309	44.3%
25-34	21	15	7		18	9	11	11		2	94	276	34.1%
35-44	6	6	3	1	11	9	3	10	1	3	53	145	36.6%
45-54	8		6		8	9	3	4	2	1	41	142	28.9%
55-64	12	6	2		5	4	2	8		1	40	190	21.1%
65+	8	14			4	12	3	17	2		60	179	33.5%
Adult(All)	77	72	36	3	65	47	26	79	9	11	425	1241	34.2%
**TB cases notified by health facility**													
B-1	5	38	37	4	55	27	17	195	12	9	399	626	63.7%
B-2	8	45	18		17	9	6	10		7	120	600	20.0%
B-3	96					1					97	343	28.3%
B-4	95		4	1	52	19	14				185	542	34.1%

EPTB – Extra pulmonary tuberculosis, Site-NOS: EPTB site not specified, LN-EXT-lymphatic extra-thoracic, LN-INT-Lymphatic intra thoracic, ABD-Abdomen, OAS – Osteoarticular spine , OAOS- Osteoarticular other than spine ,CNS-Central nervous system, DIS/MIL– Disseminated /Miliary TB.

S4E Table: Notified tuberculosis cases and extrapulmonary manifestations of tuberculosis by sex, age groups and health facilities in Federally Administered Tribal Areas, Pakistan during 2016

	Site-NOS	PLEURAL	LN-EXT	LN-INT	ABD	OAS	OAOS	CNS	DIS/MIL	OTHER	All EPTB	All TB	EPTB%
Total	7 (1.5%)	104 (22.0%)	47 (10%)	0 (0%)	235 (49.8%)	24 (5.1%)	8 (1.7%)	19 (4.0%)	1 (0.2%)	27 (5.7%)	472 (100%)	938	50.3%
Median Age (IQR)	6 (5,25)	40 (23,60)	19 (10,30)	0 (0,0)	3 (2,16)	40.5 (30,57.5)	40 (22.5,50)	25 (10,40)	40 (40,40)	27 (15,40)	18 (3,35)	24(9,42)	
**TB cases notified by sex**													
Female	1	32	22		112	8	5	7	1	18	206	440	46.8%
Male	6	72	25		123	16	3	12		9	266	498	53.4%
F:M	0.0	0.3	0.9		0.9	0.5	1.7	0.6		2.0	0.8	0.9	
**TB cases notified by age group**													
0-14	5	8	18		170	1	1	6		6	215	282	76.2%
15-24		22	10		30	1	1	3		5	72	192	37.5%
25-34	1	14	9		21	6	1	3		7	62	152	40.8%
35-44	1	14	3		7	6	2	3	1	3	40	80	50.0%
45-54		14	4		4	1	1	2		3	29	69	42.0%
55-64		10	1		2	4	1	1		2	21	74	28.4%
65+		22	2		1	5	1	1		1	33	86	38.4%
NA												3	0.0%
Adult (All)	2	96	29	0	65	23	7	13	1	21	257	653	39.4%
**TB cases notified by health facility**													
F-1	7	78	33		138	13	6	14	1	24	314	650	48.3%
F-2	0	26	14		97	11	2	5		3	158	288	54.9%

EPTB – Extra pulmonary tuberculosis, Site-NOS: EPTB site not specified, LN-EXT-lymphatic extra-thoracic, LN-INT-Lymphatic intra thoracic, ABD-Abdomen, OAS – Osteoarticular spine, OAOS- Osteoarticular other than spine, CNS-Central nervous system, DIS/MIL– Disseminated /Miliary TB

S4F Table: Notified tuberculosis cases and extrapulmonary manifestations of tuberculosis by sex, age groups and health facilities in Gilgit Baltistan, Pakistan during 2016

GB	Site-NOS	PLEURAL	LN-EXT	LN-INT	ABD	OAS	OAOS	CNS	DIS/MIL	OTHER	All- EPTB	All TB	EPTB%
**Total**	5 (1.1%)	44 (10.1%)	105 (24.0%)	(0.0%)	201 (46.0%)	31 (7.1%)	25 (5.7%)	13 (3.0%)	3 (0.7%)	10 (2.3%)	437 (100%)	1693	25.8%
**Median Age (IQR)**	26 (20,29)	25 (18,52.5)	22 (12,36)	0 (0,0)	5 (2,17)	40 (28,60)	20 (16,23)	17 (7,35)	18 (13,22)	18.5 (4,30)	17 (3,30)	18 (3,32)	
**TB cases notified by sex**													
Female	2	21	66		111	14	17	8	1	5	245	952	25.7%
Male	3	23	39		90	17	8	5	2	5	192	741	25.9%
F:M	0.7	0.9	1.7		1.2	0.8	2.1	1.6	0.5	1.0	1.3	1.3	
**TB cases notified by age group**											0.8	0.8	
0-14	1	3	31		141	1	5	5	1	4	192	735	26.1%
15-24	1	18	23		27	4	9	3	2	2	89	338	26.3%
25-34	2	7	20		15	4	5	1		3	57	200	28.5%
35-44		2	10		5	8	2	1		1	29	125	23.2%
45-54		3	8		7	2	2	1			23	102	22.5%
55-64	1	6	5		3	8	2				25	105	23.8%
65+		5	8		3	4		2			22	88	25.0%
Adult (All)	4	41	74	0	60	30	20	8	2	6	245	958	25.6%
**TB cases notified by health facility**													
G-1	3	9	14		6	1	1	1		2	37	120	30.8%
G-2	2	13	32		24	20	7	9	3	3	113	339	33.3%
G-3		14	13		80	4	4	2		3	120	347	34.6%
G-4			21		21	2	11			1	56	234	23.9%
G-5		8	25		70	4	2	1		1	111	653	17.0%

EPTB – Extra pulmonary tuberculosis, Site-NOS: EPTB site not specified, LN-EXT-lymphatic extra-thoracic, LN-INT-Lymphatic intra thoracic, ABD-Abdomen, OAS – Osteoarticular spine, OAOS- Osteoarticular other than spine, CNS-Central nervous system, DIS/MIL – Disseminated /Miliary TB

S4G Table: Notified tuberculosis cases and extrapulmonary manifestations of tuberculosis by sex, age groups and health facilities in Azad Jammu & Kashmir, Pakistan, during 2016

	Site-NOS	PLEURAL	LN-EXT	LN-INT	ABD	OAS	OAOS	CNS	DIS/MIL	OTHER	Tot.EPTB	All TB	EPTB%
**Total**	**9** (2.6%)	**136** (39.2%)	**65** (18.7%)	**1** (0.3%)	**57** (16.4%)	**26** (7.5%)	**12** (3.5%)	**13** (3.7%)	**5** (1.4%)	**23** (6.6%)	**347** 100.0%	**924**	**37.6%**
**Median Age(IQR)**	30 (30,40)	33 (24,56)	23 (14,18)	23 (23,23)	30 (18,46)	45 (35,58)	30 (24,48)	38 (17,48)	43 (22,52)	30 (22,45)	30 (21,50)	35 (21,55)	
**TB cases notified by sex**													
Female	4	48	34		32	13	7	8	3	15	164	466	35.2%
Male	5	88	31	1	25	13	5	5	2	8	183	458	40.0%
F:M	0.8	0.5	1.1	0.0	1.3	1.0	1.4	1.6	1.5	1.9	0.9	1.0	
**TB cases notified by age group**													
0-14	1	2	18		8		1	2		2	34	83	41.0%
15-24		36	18	1	16	2	2	2	2	4	83	225	36.9%
25-34	4	32	8		8	4	4	2		7	69	149	46.3%
35-44	3	13	7		10	7	1	2	1	4	48	109	44.0%
45-54		17	7		6	2	2	3	1	4	42	118	35.6%
55-64		9	2		2	10		1	1	1	26	98	26.5%
65+	1	27	5		7	1	2	1		1	45	142	31.7%
Adult (All)	8	134	47	1	49	26	11	11	5	21	313	841	37.2%
**TB cases notified by health facility**													
A-1	6	28	20	1	16	7	1	1		4	84	188	44.7%
A-2		24	3		12	4	1	3	4	9	60	174	34.5%
A-3	1	21	15		5	5	3	2		3	55	179	30.7%
A-4	2	58	25		20	9	5	6	1	7	133	310	42.9%
A-5		5	2		4	1	2	1			15	73	20.5%

EPTB – Extra pulmonary tuberculosis, Site-NOS: EPTB site not specified, LN-EXT-lymphatic extra-thoracic, LN-INT-Lymphatic intra thoracic, ABD-Abdomen, OAS – Osteoarticular spine , OAOS- Osteoarticular other than spine ,CNS-Central nervous system, DIS/MIL – Disseminated /Miliary TB

SGH Table: Notified tuberculosis cases and extrapulmonary manifestations of tuberculosis by sex, age groups and health facilities in Islamabad capital Territory, Pakistan during 2016

	Site-NOS	PLEURAL	LN-EXT	LN-INT	ABD	OAS	OAOS	CNS	DIS/MIL	OTHER	Tot.EPTB	All TB	EPTB%
Total	96 (13.5%)	174 (24.5%)	182 (25.7%)	0 (0.0%)	72 (10.2%)	47 (6.6%)	8 (1.1%)	105 (14.8%)	12 (1.7%)	13 (1.8%)	709 (100%)	1286	55.1%
Median Age (IQR)	30 (20.5,45.5)	29.5 (22,50)	30 (20,43)	0 (0,0)	30.5 (23,47)	36 (27,54)	40.5 (37,52.5)	32 (20,50)	33 (21.5,53.5)	29 (17,48)	30 (22,48)	32 (22,50)	
**SEX**													
Female	44	67	104		33	17	5	54	7	5	336	580	57.9%
Male	52	107	78		39	30	3	51	5	8	373	706	52.8%
F:M	0.8	0.6	1.3		0.8	0.6	1.7	1.1	1.4	0.6	0.9	0.8	
**AGE GROUP**													
0-14	5	6	8		2	2		9		3	35	55	63.6%
15-24	27	55	59		22	7	1	28	4	2	205	358	57.3%
25-34	21	37	39		17	12		20	2	3	151	254	59.4%
35-44	16	20	33		10	11	4	14	1		109	192	56.8%
45-54	14	20	18		10	4	1	15	2	2	86	171	50.3%
55-64	7	19	14		7	4	1	10	1	1	64	127	50.4%
65+	6	17	11		4	7	1	9	2	2	59	129	45.7%
Adults (All)	91	168	174	0	70	45	8	96	12	10	674	1231	54.8%
**Health Facility**													
I-1	96	174	179		71	47	8	105	11	10	701	1256	55.8%
I-2			3		1				1	3	8	30	26.7%

EPTB – Extra pulmonary tuberculosis, Site-NOS: EPTB site not specified, LN-EXT-lymphatic extra-thoracic, LN-INT-Lymphatic intra thoracic, ABD-Abdomen, OAS – Osteoarticular spine, OAOS- Osteoarticular other than spine, CNS-Central nervous system, Dis/Mil – Disseminated /Miliary TB
