# Supplementary material for: Extrapulmonary tuberculosis in Pakistan- A nation-wide multicenter retrospective study
Source: PLoS One. 2020 Apr 28;15(4):e0232134. doi: 10.1371/journal.pone.0232134 (PMC7188211; doi:10.1371/journal.pone.0232134)
Supplement: Attachment — Submitted filename: Respone to reviewers.pdf [file pone.0232134.s006.pdf]

Dear editor,

We wish to thank you for very thorough and constructive comments on our manuscript entitled, “**Extrapulmonary tuberculosis in Pakistan- A Nation-wide Multicenter Retrospective study**" (Original Article)

We have revised the manuscript based on your comments. Here we provide point-to-point replies. The changes in the paper are marked in red color in the resubmitted manuscript.

Comments to the Author

1. Please ensure that your manuscript meets PLOS ONE's style requirements, including those for file naming.

We have now edited the manuscript according to style template given at http://www.journals.plos.org/plosone/s/file?id=wjVg/PLOSOne_formatting_sample_main_body.pdf

2. We note that Figure S1 in your submission contains map images which may be copyrighted.

We have now obtained permission from the original copyright holder of Figure S1 to publish the content specifically under the CC BY 4.0 license. Source and caption of the figure is added with the figure

Content permission on PLOSONE recommended format. Three file are uploaded named as “Other1” approval on PLOSONE recommended format, “Other2” email communication and “Other3” terms and condition of D-maps.com

3. Please amend your list of authors on the manuscript to ensure that each author is linked to an affiliation. Authors’ affiliations should reflect the institution where the work was done (if authors moved subsequently, you can also list the new affiliation stating “current affiliation:….” as necessary).

Thank you for your guidance we have now edited authors' affiliation as recommended in http://www.journals.plos.org/plosone/s/file?id=ba62/PLOSOne_formatting_sample_title_authors_affiliations.pdf

Review Comments to the Author

Reviewer #1: Authors have chosen a relevant topic. Current manuscript is technically sound and relevant to the subject concerned. Study covers large sample size which provides insight into demography and clinical manifestation of extra pulmonary cases.

Thank you very much

Reviewer #2: The manuscript by Tahseen et. al, has provided some insight into epidemiology of extrapulmonary tuberculosis (EPTB) in Pakistan. Despite various limitations, authors have tried to study the prevalence of EPTB across various regions, genders and age groups in Pakistan. 'EPB' word in the abstract needs to be corrected as 'EPTB'. It would also be important for the reader to know about the possible reasons behind the association of specific risk factors (sex, race, ethnicity and provenance) with epidemiology of EPTB. Viewpoints of the authors regarding such correlation with appropriate references are lacking in this manuscript. I think the authors should very critically discuss their findings under the discussion part. A thorough discussion will complement the limited data availability in this study. This would also tremendously increase the value of the manuscript.

Thank you very much for your comment, we have now edited the discussion section and critically discussed the key findings and added our view points.

<u>Changes in manuscript;</u>

- All changes are marked in red color in the resubmitted manuscript.
- Some text is rearranged in results section to better align with edits in discussion without changing the content.
- Colored figures (Figure 1 and 2) uploaded to replace black and white, uploaded originally with manuscript for more clarity without any change in the content
- Support table: Table S4A-H: In all tables an additional row is added under age group - "Adults (All)" for cumulative number for adult of all age groups .
